# The importance of debunking constructions

**DOI:** 10.1177/14550725221150346

**Published:** 2023-01-25

**Authors:** Matilda Hellman

**Affiliations:** University of Helsinki, Helsinki, Finland

The great majority of the work published in addiction journals stems from the Anglophone world (Hellman et al., 2020). In *Nordic Studies on Alcohol and Drugs (NAD)* we have discussed what the dominance of the English language in academia means from a Nordic perspective. There is some risk that the concepts and terminology of importance for the Nordic region become lost in translation. (Andersen & Hellman, 2021). In this issue, the debate is taken further in a position paper by Bahji and colleagues (2023) on how and why scientific contributions from the non-English-speaking world have been excluded from addiction literature. They also suggest how the non-Anglophone literature could be made more accessible.

The question of language use and concept choices cannot be stressed enough: how we understand and communicate the world is through constructs, through language conventions influenced by epistemic cultures. Our understanding of alcohol, drugs, tobacco and gambling looks different and appears different in different languages and geographies.

Constructionism or social constructionism as a social science approach is usually dated to the 1960s, and more specifically to Peter L Berger and Thomas Luckman's influential work, *The social construction of reality* (1966). All perception of reality – whether it concerns our everyday life, society or ourselves – is organised in constructions, Berger and Luckman argue. At the time of the book's publication, the path was already well paved by the critical theorists, especially those adhering to Marxism and the Frankfurt School, who embraced reflective critical analysis to examine society and culture. A typical focus of social constructionism is the institutionalisation and internalisation of everyday beliefs and knowledge.

Today, the notion of constructions or constructs is widely accepted and normalised due to a general awareness of humans’ cognitive and symbolic meaning-making. Humans arrange their observations and comprehensions in certain ways for the world to make sense. In this issue of the *NAD*, Bærndt and Frank (2023) discuss the ways in which young Muslim women reflect upon themselves as part of Danish alcohol culture. The authors delve into the women's meaning-making of how others understand and categorise them in relation to certain (social) constructions. These young women in Denmark tone down being a Muslim to avoid negative comments based on stereotypes of Muslims and their drinking. Identifying as both Muslim and Danish leads to several of the young women experiencing an identity crisis.

Crucially, we need to pick apart constructions because they are not relative in all infinity, like fairy tales, dreams or fantasies. The concept of construction includes the idea that in one way or another it sets out to represent reality according to certain assumptions of (human) reason and understanding. At best, constructions serve to bring more honesty in knowledge-seeking attempts. However, it hardly comes as a surprise that constructions may contain distorted or harmful views from a societal perspective. In their contribution, Lindeman and colleagues (2023) inquire into the ways in which alcohol, food and gambling operators address the public on social media. Their analysis of a substantial data set (N = 13,241) of posts published by Finnish and Swedish operators on YouTube, Twitter, Facebook and Instagram between 2017 and 2020 shows that most of the posts do not thematically or in any obvious way relate to gambling or games. In the Swedish licence system, operators seem to present themselves more straightforwardly as gambling companies, whereas in the Finnish monopoly system the image is more tied to a social role of a public benefactor. This is a social construction that justifies the state gambling monopoly in Finland. As in political image building, the constructions aimed to enhance company reputation are ordered in value-laden stories. They can remain invisible if they are not continuously named, noticed and questioned.

## Also in this issue

El-Guebaly and colleagues (2023) discuss the critical role of peer reviewers as gatekeepers in the addiction publication sector. Belfrage and colleagues (2023) have studied traumatic experiences and symptoms of posttraumatic stress disorder in substance use disorder. Leventelis and colleagues (2023) report on the development and validation of the pandemic medication-assisted treatment questionnaire.
